# Lesions of the Orbitofrontal but Not Medial Prefrontal Cortex Affect Cognitive Judgment Bias in Rats

**DOI:** 10.3389/fnbeh.2017.00051

**Published:** 2017-03-21

**Authors:** Joanna Golebiowska, Rafal Rygula

**Affiliations:** Affective Cognitive Neuroscience Laboratory, Department of Behavioral Neuroscience and Drug Development, Institute of Pharmacology, Polish Academy of SciencesKrakow, Poland

**Keywords:** rat, cognitive judgment bias, orbitofrontal cortex, medial prefrontal cortex (mPFC), lesion

## Abstract

Neuroimaging studies in humans have recently shown that the prefrontal cortex (PFC) and orbitofrontal cortex (OFC) mediate bias in the judgment of forthcoming events. In the present study, we sought to determine whether cognitive judgment bias (CJB) is also dependent on these prefrontal regions in non-human animals. For this, we trained a cohort of rats in the ambiguous-cue interpretation (ACI) paradigm, subjected them to excitotoxic lesions in the medial PFC (mPFC) and OFC, and tested the effects of neuronal loss within these regions on CJB. Comparison of the lesions’ behavioral effects in the ACI paradigm revealed that neuronal loss within the OFC but not within the mPFC influences the interpretation of ambiguous cues by animals. Our findings demonstrate the specific involvement of the OFC in CJB in rats.

## Introduction

For some, the glass is always half empty, while for others, it is always half full. These biases in cognitive judgment, termed pessimism and optimism, respectively, influence many aspects of human life and have been implicated in the etiology and maintenance of several psychiatric disorders, including depression and mania (Brittlebank et al., 1993; Nygren et al., 1996; Murphy et al., 1999). In humans, cognitive judgment of forthcoming events is biased mainly by emotions. Specifically, negative emotions have been linked with pessimistic tendencies in interpretation of ambiguous stimuli and with increased expectation of punishment (Murphy et al., 1999, 2003). Conversely, positive affective states have been linked with optimistic judgments (Nygren et al., 1996). The same is becoming evident for animals. Since the publication of a pioneering study by Harding et al. (2004) demonstrating that rats subjected to stress show “pessimistic” judgment bias, the existence of cognitive biases in animals have been described in a range of species, following different behavioral manipulations (Bethell et al., 2007; Enkel et al., 2010; Mendl et al., 2010; Bateson et al., 2011; Doyle et al., 2011; Bethell and Koyama, 2015). However, very few studies to date have attempted to characterize the potential neurochemical and neuroanatomical correlates of optimistic or pessimistic cognitive judgment bias (CJB). In 2012, the first potential neurochemical substrate for optimism was identified. Sharot et al. (2012) demonstrated that administration of a drug that enhances dopaminergic function (dihydroxy-L-phenylalanine; L-DOPA) increases optimism in humans by reducing negative expectations regarding the future. In this study participants treated with L-DOPA but not with selective serotonin reuptake inhibitor—citalopram, showed enhanced learning from desirable information regarding the future and decreased learning from undesirable information what resulted in optimistic bias. Previously, using functional magnetic resonance imaging (fMRI), the same authors found increased activation (the blood-oxygenation-level-dependent (BOLD) signal) in several brain regions including the anterior cingulate cortex (ACC), the ventral and the dorsal medial prefrontal cortex (mPFC) and the amygdala (AMYG) when participants imagined that events in the future will be positive compared with when they imagined that they will be negative (Sharot et al., 2007). Moreover, across individuals, activity in the rostral ACC was correlated with trait optimism (Sharot et al., 2007). Despite these findings, information about the neuroanatomical underpinnings of (CJB) remains scarce.

In the present study, we sought to determine the functional contributions of two distinct regions of the prefrontal cortex (PFC) to CJB in rats. For this, we subjected the animals to selective excitotoxic lesions of the medial prefrontal and orbitofrontal cortices and compared the behavioral effects of these manipulations by using the ambiguous-cue interpretation (ACI) test. In this behavioral paradigm, the rats were trained to press a lever in an operant chamber to avoid punishment by mild electric foot shock and to press another lever in response to a different tone to receive a food reward. The tones acquired negative and positive valence, and the animals were trained until they achieved a stable, correct performance in discriminating between them. During the ambiguous-cue testing the animals were presented with an additional tone at a frequency that was intermediate between the positive and negative tones. The pattern of lever presses in response to this intermediate (ambiguous) tone was recorded and taken as an indicator of the valence of rat’s expectation (optimism/pessimism; for details, see Enkel et al., 2010; Rygula et al., 2012, 2013).

We hypothesized that neuronal loss within distinct regions of the PFC would differentially affect the animals’ interpretation of the ambiguous cue.

## Materials and Methods

### Ethics Statement

All described experiments were approved by the Ethics Committee for Animal Experiments at the Institute of Pharmacology Polish Academy of Sciences and were conducted in accordance with the NIH Guide for the Care and Use of Laboratory Animals.

### Subjects and Housing

In this study we used 34 male Sprague-Dawley rats (Charles River, Germany) that weighed 175–200 g upon arrival. The rats were housed in groups of four, in standard type IV macrolon cages (59 × 38 × 20 cm) in a temperature (21 ± 1°C) and humidity (40%–50%)-controlled room under a 12/12-h light/dark cycle (lights on at 07:00 h). The experimental procedures and testing were performed during the light phase of the light/dark cycle, between 08:00 h and 16:00 h. From 1 week prior to training and during all of the experiments, the rats were subjected to food restriction to approximately 85% of their free-feeding weights. This goal was achieved by providing 15–20 g of food per rat per day (standard laboratory chow). Water was available *ad libitum*.

### Apparatus

The behavioral tasks were performed in eight computer-controlled operant conditioning boxes (MedAssociates, St. Albans, VT, USA), where each box was equipped with light, a speaker, a liquid dispenser (set to deliver 0.1 ml of 5% sucrose solution), a grid floor through which scrambled electric shocks (0.5 mA) could be delivered, and two retractable levers. The levers were located on opposite sides of the feeder. All of the behavioral protocols were programmed using the Med State notation code (Med Associates).

### Behavioral Training for the ACI Paradigm

All the experimental training and testing procedures for the ACI paradigm used in this study have been described in detail previously (Enkel et al., 2010; Rygula et al., 2012, 2015b). The training consisted of three phases: positive tone training, negative tone training and discrimination training.

#### Positive Tone Training

During this phase, we trained the rats to press the lever located on the left side of the feeder to receive sucrose solution when certain tones (50 s, 9000 Hz at 75 dB or 50 s, 2000 Hz at 75 dB counterbalanced) signaled the availability of a reward. Because of their association with a palatable sucrose solution, these tones acquired a positive valence and were referred to as the “positive” tones, and the associated lever was referred to as the “positive” lever. Positive tone training consisted of three training steps that used three different training protocols: step (a) presentations of the positive tones (each lasting 10 s) co-occurred with the delivery of the sucrose solution and were separated by 10-s intertrial intervals (ITIs); step (b) presentations of the positive tones (each lasting 50 s), co-occurred with left lever extensions and were followed by 10-s ITIs (each lever press during the tone was rewarded by the delivery of the sucrose solution (liquid dipper arm lifted for 10 s)); and step (c) a procedure similar to (b), however the first lever press in a given trial initiated the reward delivery, retracted the lever, terminated the tone and initiated the ITI. Each training session lasted for 30 min. There was no quantifiable measure of performance during step (a), but the training was repeated until the rats learned the location of the reward and spent most of their time with their heads in the liquid dipper aperture. During step (b) the training sessions continued until the rats attained a stable performance of more than 200 responses maintained over three consecutive training sessions. During step (c) the training continued until the animals reached a minimum of 90% successful responses to the positive lever following positive tones presentations maintained over three consecutive training sessions. Positive tone training was followed by negative tone training.

#### Negative Tone Training

During this phase, we trained the rats to press a lever that was located on the right side of the feeder to avoid an electric shock (0.5 mA, 10 s) when tones (50 s, 2000 Hz at 75 dB or 50 s, 9000 Hz at 75 dB counterbalanced) signaled a forthcoming punishment. Because of their association with a concomitant punishment, these tones acquired a negative valence and were referred to as the “negative” tones. The associated lever was referred to as the “negative” lever. Negative tone training consisted of two training steps. During step (a) the presentations of the negative tones were accompanied by the occurrence of electric shocks unless the rat pressed the right (negative) lever, which terminated the shock and tone presentation and initiated a 30 s ITI. During step (b) the presentations of the negative tones preceded the occurrence of the electric shocks. The delay from the tone onset to the electric shock occurrence was progressively increased from 1 s to 40 s. Pressing the negative lever before the shock onset, referred to as the “prevention” response, terminated the tone, prevented forthcoming punishment and began a 10 s ITI. Pressing the negative lever after the shock onset terminated the tone and shock and was referred to as the “escape” response. The maximum duration of the tone/shock application was 50 s (i.e., 40 s of tone presentation followed by 10 s of a tone/shock co-occurrence), and the tone presentations were separated by 10 s ITIs. During the ITIs, the levers retracted. Daily training sessions contained 40 tone presentations. The animals were required to accomplish at least 60% correct “prevention” responses maintained over three consecutive training sessions before they were allowed to proceed to the discrimination training. The performance criterion for the “prevention responses” was set lower than that for the reward responses because during the negative tones training, the animals had to additionally overcome their natural tendency to freeze after the presentation of the conditioned stimulus (negative tone). In the present study the rats were always trained in the following order: positive tone training, negative tone training and discrimination training. Although the order of the fist two training steps could have some influence on behavior of the animals in the ACI test, this is unlikely, and in our opinion was eliminated during the discrimination phase, when the rats were presented with both tones in random order until they have reached the criterion of at least 70% of correct responses to each tone.

#### Discrimination Training

During this phase, the rats were trained to discriminate between positive and negative tones by responding to positive and negative levers (as learned previously) to minimize punishment and maximize reward delivery. The tones, (20 positive and 20 negative), were presented in pseudo-random order (pre-programmed sequence: 0, 1, 0, 1, 0, 1, 1, 0, 0, 1, repeated 4×, where 0 = a positive and 1 = a negative tone) with 10 s ITIs (levers retracted). Pressing the positive lever during the positive tone presentation resulted in an instant reward delivery and initiated the ITI. Pressing the negative lever during the negative tone presentation resulted in termination of the negative tone, prevented forthcoming punishment and initiated the ITI. Pressing the wrong lever (e.g., pressing the right lever instead of the left lever in response to a positive tone presentation) and “escape” responses or response omissions were considered failed trials. Rats had to reach the criterion of a minimum of 70% correct responses for each lever, maintained over three consecutive training sessions, to qualify for ACI testing. This criterion has been established based on seminal experiment by Enkel et al. (2010) and was validated in our previous experiments as sufficient for detecting changes in CJB in rats (Rygula et al., 2012, 2013, 2014a, 2015a,b; Drozd et al., 2016; Rafa et al., 2016).

### Ambiguous-Cue Testing

The ACI testing session consisted of 50 (20 positive, 20 negative, and 10 intermediate (ambiguous)) tone presentations. We set the frequency of the intermediate tones to 5000 Hz at 75 dB on the basis of the protocol described by Enkel et al. (2010) and our own pilot experiments (data not shown). The tones were presented in a pre-programmed pseudo-random order (0, 2, 0, 1, 2, 0, 0, 2, 1, 2 repeated 5×, where 0 = positive, 1 = ambiguous and 2 = negative tone) and were separated by 10 s ITIs, during which the levers were retracted. Any lever press during the ambiguous tone presentation terminated the tone but had no consequences. If the rat did not respond within 50 s of the ambiguous tone presentation, the tone was terminated and a response omission was scored. To evaluate the effects of excitotoxic lesions within the orbitofrontal cortex (OFC) and mPFC on cognitive bias of rats, the responses to each tone (positive, ambiguous and negative) during ACI testing were scored and analyzed as the proportion of the overall number of a given tone presentations (including response omissions). To calculate the CJB index (CJBI), we subtracted the proportion of negative responses to the ambiguous cues from the proportion of positive responses to the ambiguous cues, which resulted in values ranging between −1 and 1. Values above 0 indicated an overall positive judgment and “optimistic” interpretation of the ambiguous cue, while the values below 0 indicated overall negative judgment and “pessimism”. The proportion of omissions was analyzed separately.

### Surgical Procedures

Cytoarchitectonic divisions of the OFC and mPFC were defined according to the stereotaxic atlas of Paxinos and Watson (1998). The rats were anesthetized by intramuscular injection (i.m.) of 100 mg/kg ketamine (Biowet, Pulawy, Poland) and 10 mg/kg xylazine (Sedazin Biowet, Pulawy, Poland) and were placed in a stereotaxic head holder (RWD Life Science, San Diego, CA, USA) fitted with atraumatic ear bars. The scalp was retracted, and holes were drilled into the skull to expose the target regions of the brain. Bilateral excitotoxic lesions in the OFC and mPFC were created using an infusion of 0.18 M quinolinic acid (Sigma-Aldrich, Poznan, Poland) in physiological saline (brought to a neutral pH with 1 M NaOH) through a 30-gauge stainless steel cannula connected via polyethylene tubing to a 1 ml glass Hamilton syringe in a microdrive pump. The quinolinic acid has been selected based upon its effectivity in cortical lesions supported by our own laboratory experience and literature data (e.g., Mar et al., 2011). Lesion coordinates and toxin volumes for the OFC were anterior (A) = +4.0 mm to bregma, lateral (L) = ±0.8 mm to the midline, and vertical (V) = −3.4 mm from the dura (0.4 μL); (A) = +3.7 mm, (L) = ±2.0 mm, and (V) = −3.6 mm (0.6 μL); and (A) = +3.2 mm, (L) = ±2.6 mm, and (V) = −4.4 mm (0.4 μL). Lesion coordinates and toxin volumes for the mPFC were (A) = +3.5 mm to bregma, (L) = ±0.7 mm to the midline, and (V) = −4.5 mm from dura (0.8 μL) and (A) = +2.5 mm, (L) = ±0.7 mm, and (V) = −4.5 mm (0.8 μL). The incisor bar was set −3.3 mm below the interaural line. Infusions were made at a rate of 0.25 μL/min, with a further 3 min allowed for diffusion before the cannula was retracted, and the wound was cleaned and sutured. Sham-operated rats received identical infusions of physiological saline. Postoperatively, all rats were given 1 mg/kg i.m. of meloxicam (Metacam, Boehringer Ingelheim, Vetmedica GmbH, Germany).

### Experimental Design

**Figure 1:**
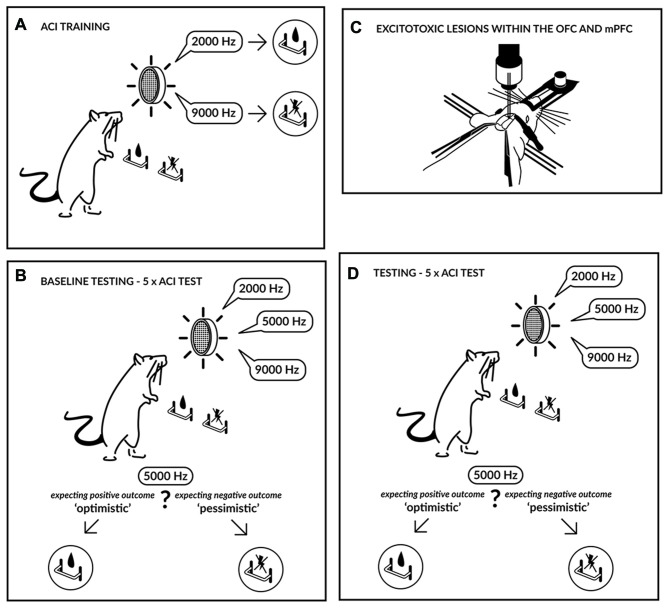
**Experimental schedule.** Initially the rats were trained **(A)** to press a lever in an operant conditioning chamber to receive a sucrose-solution reward that was contingent on one tone (2000 Hz) and to press another lever in response to a different tone (9000 Hz) to avoid punishment by a mild electric foot-shock. The tones, which served as discriminative stimuli, acquired either a positive or negative valence, and the training continued until the rats accomplished a stable, correct discrimination ratio. After attaining a stable discrimination performance, the animals were evaluated in the ambiguous-cue interpretation (ACI) test **(B)**. The ACI test was composed of a discrimination task, as described above, but included the presentation of additional tones with intermediate frequencies (5000 Hz). The lever-press response pattern to these ambiguous cues was considered an indicator of the rat’s expectation of a positive or negative event; in other words, it represented a positive/“optimistic” or a negative/“pessimistic” bias. Following baseline testing, the animals were subjected to excitotoxic lesions within the medial prefrontal cortex (mPFC) or orbitofrontal cortex (OFC) **(C)** and were subsequently re-tested using the ACI test **(D)**.

The experimental design is shown in Figure 1. Trained animals were subjected to five consecutive ACI tests at 2-day intervals (baseline). Based on their baseline performance, the animals were assigned to three experimental groups based on their average CJBI. These three groups of rats were subjected to excitotoxic lesions in either the OFC (*N* = 13) or the mPFC (*N* = 14) or to SHAM surgery (*N* = 12; 5 mPFC and 7 OFC SHAM-operated animals) and, after a 1-week recovery period, were tested on discrimination of the positive and negative reference tones in once-daily sessions. ACI testing was not initiated until all SHAM-operated animals regained their pre-surgery performance in response to the positive and negative reference tones (minimum of 70% correct responses for each lever, maintained over three consecutive training sessions). After this time point any deficits in responding to reference tones in the OFC or PFC lesioned animals were treated as “lesion-induced”.

To assess the effects of lesions on CJB, after the recovery period, the animals were re-evaluated in five consecutive ACI tests carried out at 2-day intervals (test). After behavioral testing was completed, the rats were decapitated, and their brains were removed, frozen and cut using a freezing microtome. Coronal sections (60 μm) were stained with Cresyl Violet, visualized with a microscope under conventional bright field illumination, and photographed digitally. The location of the lesions was mapped onto a standardized atlas of the rat brain (Paxinos and Watson, 1998).

### Statistics

The data were analyzed using SPSS (version 21.0, SPSS Inc., Chicago, IL, USA). The effects of SHAM, OFC and mPFC lesions on the CJBI were investigated using a two-way repeated-measures analysis of variance (ANOVA) with the within-subjects factor test (2 levels: baseline and post-surgery) and the between-subjects factor lesion (3 levels: SHAM, OFC and mPFC). The effects of the lesions on the proportion of responses via each lever following the presentation of ambiguous cues and reference tones were investigated using four-way repeated-measures ANOVA with the within-subjects factors test (2 levels: baseline and post-surgery), lever (2 levels: positive and negative) and tone (3 levels: positive, ambiguous and negative) and the between-subjects factor lesion (3 levels: SHAM, OFC and mPFC). Response omissions were analyzed separately using two-way repeated-measures ANOVA with the within-subjects factors test (2 levels: baseline and post-surgery) and tone (3 levels: positive, ambiguous and negative) and the between-subjects factor lesion (3 levels: SHAM, OFC and mPFC). For pairwise comparisons, the values were adjusted using Sidak’s correction factor for multiple comparisons (Howell, 1997). All of the tests of significance were performed at α = 0.05. For repeated-measures analyses, the sphericity was verified using Mauchly’s test. The data are presented as the mean ± SEM.

## Results

**Figure 2:**
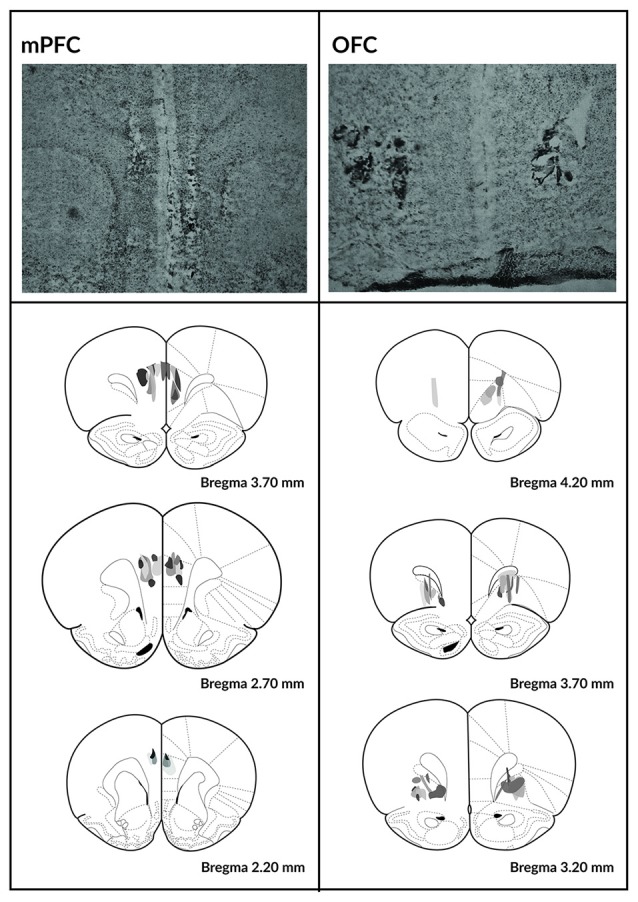
**Histological analysis of the mPFC and OFC lesions.** Photomicrographs depicting typical lesions in the mPFC (top left) and OFC (top right) and schematics depicting lesion assessments for the mPFC (bottom left) and OFC (bottom right). Grayscale shading indicates the extent of neuronal loss across subjects, with each subject represented as a separate, stacked layer. Diagrams are modified from Paxinos and Watson (1998).

Histological verification of the size and location of the lesions is depicted in Figure 2. Two animals from the SHAM-operated group were excluded because their responses never recovered following the surgery. Three animals from the OFC lesion group and three from the mPFC lesion group were excluded because the lesions could not be localized.

### Effects of Excitotoxic Lesions in the Medial Prefrontal and Orbitofrontal Cortices on Rat Cognitive Judgment Bias in the ACI Test

**Figure 3:**
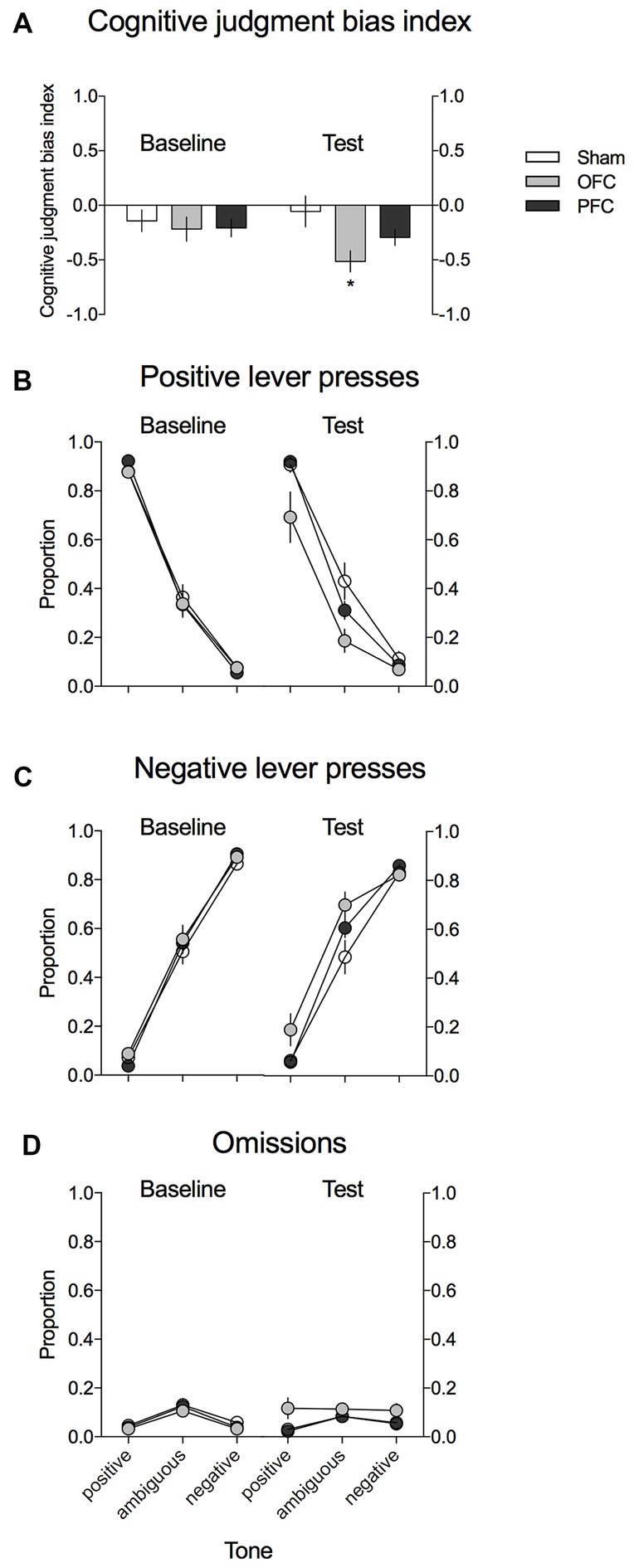
**Effects of the excitotoxic lesions in the mPFC and OFC on cognitive judgment bias (CJB) in rats, using the ACI paradigm.** **(A)** CJB index (CJBI) of the SHAM-operated rats (white bars) and rats with excitotoxic lesions within the OFC (gray bars) and mPFC (black bars), measured using the ACI paradigm before and after the surgery. **(B)** Proportion of positive, **(C)** proportion of negative, and **(D)** proportion of omitted responses to the trained and ambiguous tones in the control (white circles) and rats with excitotoxic lesions within the OFC (gray circles) and mPFC (black circles), measured using the ACI paradigm before and after the surgery. *Indicates statistically significant (*p* < 0.05) differences between the SHAM and OFC-lesioned animals and statistically significant (*p* < 0.05) differences between the baseline and test in the OFC-lesioned animals. Data are presented as the mean ± SEM.

As shown in Figure 3A, the CJBI did not differ between the experimental groups in the baseline ACI tests. After the surgery and recovery period, the CJBI was significantly lower in animals with excitotoxic lesions within the OFC than in SHAM-operated controls (*p* = 0.016) or at baseline (*p* = 0.002). The CJBI of mPFC-lesioned animals did not differ significantly from the baseline or from the SHAM-operated controls (Figure 3A). Two-way repeated-measures ANOVA revealed statistically significant effects of test (*F*_(1,28)_ = 4.29, *p* = 0.048) and of the test × lesion interaction (*F*_(2,28)_ = 5.17, *p* = 0.012).

As shown in Figures 3B,C, this negative/“pessimistic” response bias observed in rats after the excitotoxic OFC lesion was evidenced by the significantly decreased proportion of positive lever presses and the significantly increased proportion of negative lever presses in response to the ambiguous tone relative to SHAM-operated controls (*p* = 0.01 and *p* = 0.039, respectively) and to the baseline (*p* = 0.001 and *p* = 0.02, respectively). Four-way repeated-measures ANOVA revealed a significant test × lesion × lever interaction (*F*_(2,28)_ = 6764, *p* = 0.004).

Analysis of the proportion of trials in which the animals did not respond (Figure 3D) revealed that following the OFC lesions, the rats made significantly more omissions (*p* = 0.015) than during the baseline assessment and more omissions (statistical trend: *p* = 0.062) than their SHAM-operated conspecifics. Three-way repeated-measures ANOVA revealed a significant test × lesion interaction (*F*_(2,28)_ = 3895, *p* = 0.032).

## Discussion

In the present study, lesions within the OFC but not within the mPFC decreased the ratio of positive lever presses and increased the ratio of negative lever presses in response to the ambiguous and positive experimental tones, rendering the rats more “pessimistic”. These results reveal the involvement of the OFC in the modulation of CJB in rats and provide insights into the distinct contributions of different regions of the PFC in decision-making processes under ambiguity.

Our finding that neuronal loss within the OFC altered the CJB of rats was not surprising, especially considering that previous studies with both humans and non-human animals have implicated this region in the processing of both present and expected reinforcement cues (Schoenbaum et al., 1999; Tremblay and Schultz, 1999; Schultz et al., 2000). Single-cell studies have directly shown that cells in the OFC fire in anticipation of appetitive and aversive reinforcers and revealed that the OFC also encodes the relative values of stimuli (Nobre et al., 1999; Tremblay and Schultz, 1999). Increased activity within the OFC has also been consistently associated with processing of positive affect, such as the encoding of reward, value and pleasure (Kringelbach, 2005; Grabenhorst and Rolls, 2011), approach-oriented processing (Eddington et al., 2007) and sensitivity to environmental changes (Kringelbach, 2005). These findings suggest that activity in the OFC contributes to an optimistic attitude (Flagan and Beer, 2013). Conversely, OFC hypo activity resulting from neuronal loss might cause pessimism.

Another interpretation of the pessimistic judgment bias observed following the OFC lesions is decreased risk-taking behavior in the rats, which may have resulted in a higher proportion of “safe” or “pessimistic” choices of the lever associated with avoiding punishment. This explanation is consistent with the results of a study by Orsini et al. (2015), which demonstrated that OFC lesions decrease risky choices. However, these results conflict with other preclinical and clinical data showing that rodents and humans with OFC damage undertake more risk in laboratory gambling and probability discounting tasks (Rogers et al., 1999; Bechara, 2001; Pais-Vieira et al., 2007; Stopper et al., 2014). These discrepancies may exist because the risk involved in the latter studies was limited to omission of a reward or loss of points in a token economy, whereas in our study and in the study by Orsini et al. (2015), the risk involved real punishment.

Finally, the association between pessimistic judgment bias and the neuronal loss within the OFC observed in our study is consistent with previous structural and functional neuroimaging human studies demonstrating associations between OFC gray matter volume, anxiety and levels of optimism (Dolcos et al., 2016) and between smaller OFC volume and increased negative affect (Ansell et al., 2012). In the latter study, cumulative lifetime adversity was linked with smaller gray matter volume in key frontal brain regions, including the ventromedial and OFC.

Although the ACI paradigm used in our study does not allow us to establish the precise neural mechanism underlying the observed negative/“pessimistic” judgment bias, the deficit in processing of positive stimuli agrees with other accounts of OFC function emphasizing the role of this region in the representation of information regarding reinforced outcomes, response selection, and response inhibition (Rolls, 2000). Performing the ACI task is expectancy-guided and requires learned associative access to specific representations of the two reinforced outcomes (reward and punishment) following auditory cues (reference tones), the selection of an instrumental response (positive or negative lever) and the suppression of the response inappropriate to the expected outcome. Thus, in principle, disturbance in any of these processes could underlie the OFC lesion-induced effects in the ACI task.

Notably, decreased positive responses following OFC lesion were not limited to ambiguous tones but also generalized to trained, positive reference tones. This observation constitutes interesting although not entirely new phenomenon. Similar effects were observed in the seminal study by Enkel et al. (2010) following stress-mimicking (reboxetine + corticosterone) pharmacological treatment and in some of our previous studies (e.g., Rygula et al., 2014b) following mazindol treatment). The proposed explanation for this phenomenon implies that the OFC lesion decreased the positive associations with the tones. Indeed, the pattern of omissions, which were significantly increased in response to positive tones, supports this view and suggests decreased motivation to respond to positive cues but not to negative cues. The general motivational deficits seem however unlikely, given that the response rates did not decrease for the negative lever. Another explanation could imply the OFC lesion induced inability to inhibit fear response. Indeed, as demonstrated by Zelinski et al. (2010), lesions to the OFC but not infralimbic/prelimbic PFC lead to a generalized fear response and impaired extinction. The absence of OFC-mediated inhibitory functions could have led to an uninhibited fear response, and in consequence, to a decreased positive responding in the ACI task.

In our study, neuronal loss within the mPFC did not affect the behavior of rats in the ACI paradigm. Animals with the mPFC lesions pressed the positive and negative levers in response to the ambiguous cue as they did prior to surgery and similar to the sham-operated control rats. This result was surprising considering the previous studies that have implicated the mPFC in tracking variations in probability of a reward (St Onge and Floresco, 2010; St Onge et al., 2012; Orsini et al., 2015) but is consistent with the recent finding by Dalton et al. (2016) that inactivation of the mPFC does not impair reversal learning with probabilistic outcomes and with the findings of Sul et al. (2010), who investigated, in a dynamic two-armed-bandit task, how value-based decision-making is mediated by different subregions of rodent PFC. In that study, both the orbitofrontal and medial prefrontal cortices conveyed signals related to the outcomes of animals’ past choices, but neural signals for reward prediction errors were processed only in the OFC.

As the lesions in the mPFC in our study were relatively discrete and mainly localized within the prelimbic regions, it is possible that lesions encompassing a larger portion of the mPFC and including, for example, the ACC might elicit stronger effects on CJB in tested animals. Indeed, neuroimaging studies in humans identified the rostral portion of the ACC as one of the neuroanatomical correlates of optimistic bias in humans (Sharot et al., 2007). We cannot also exclude the possibility that mPFC lesions were simply too small to elicit effects on behavior of animals in the ACI test. Further studies with larger lesions and lesions targeting separate parts of the mPFC (infralimbic, prelimbic or cingulate) will provide more definitive answers about the involvement of separate fronto-cortical regions in CJB in rats.

## Conclusion

Using the recently developed ACI test, we demonstrated for the first time that neuronal loss within the OFC but not mPFC affects CJB in rats. Our findings provide novel information regarding the involvement of two different prefrontal regions in mediating decision-making under ambiguity. These results also provide insight into the possible neuroanatomical correlates of biased judgments observed in affective disorders such as depression or mania.

## Author Contributions

JG and RR conceived and designed the experiments; read the manuscript and provided critical feedback. JG performed the experiments. RR provided materials and analysis tools; wrote the manuscript.

## Funding

This work was supported by the Polish National Science Centre (Research grant: 2012/07/E/NZ4/00196 to RR) and the statutory funds of the Institute of Pharmacology, Polish Academy of Sciences. Publication charge was supported by KNOW funds MNiSW-DS- 6002-4693- 26/WA/12.

## Conflict of Interest Statement

The authors declare that the research was conducted in the absence of any commercial or financial relationships that could be construed as a potential conflict of interest.
